# Convergence of soil nitrogen isotopes across global climate gradients

**DOI:** 10.1038/srep08280

**Published:** 2015-02-06

**Authors:** Joseph M. Craine, Andrew J. Elmore, Lixin Wang, Laurent Augusto, W. Troy Baisden, E. N. J. Brookshire, Michael D. Cramer, Niles J. Hasselquist, Erik A. Hobbie, Ansgar Kahmen, Keisuke Koba, J. Marty Kranabetter, Michelle C. Mack, Erika Marin-Spiotta, Jordan R. Mayor, Kendra K. McLauchlan, Anders Michelsen, Gabriela B. Nardoto, Rafael S. Oliveira, Steven S. Perakis, Pablo L. Peri, Carlos A. Quesada, Andreas Richter, Louis A. Schipper, Bryan A. Stevenson, Benjamin L. Turner, Ricardo A. G. Viani, Wolfgang Wanek, Bernd Zeller

**Affiliations:** 1 Division of Biology, Kansas State University, Manhattan, KS, 66506, USA; 2 Appalachian Laboratory, University of Maryland Center for Environmental Science, Frostburg, MD, 21532, USA; 3 Department of Earth Sciences, Indiana University-Purdue University, Indianapolis, IN, 46202, USA; 4 UMR 1220 TCEM, INRA, Bordeaux Sciences Agro, Villenave d'Ornon, 33883, France; 5 National Isotope Centre, GNS Science, Lower Hutt, 5040, New Zealand; 6 Department of Land Resources and Environmental Sciences, Montana State University, Bozeman, MT, 59717, USA; 7 Department of Biological Sciences, University of Cape Town, Rondebosch, 7701, South Africa; 8 Forest Ecology and Management, Swedish University of Agricultural Sciences (SLU), Umeå, 90183, Sweden; 9 Earth Systems Research Center, Morse Hall, University of New Hampshire, Durham, NH, 03824, USA; 10 Departement of Environmental Sciences - Botany, Schönbeinstrasse 6, 4056 Basel Switzerland; 11 Institute of Agriculture, Tokyo University of Agriculture and Technology, Fuchu, Tokyo, 1838509, JAPAN; 12 British Columbia Ministry of Forests, Lands and Natural Resource Operations, Victoria, British Columbia, V8Z 5J3, Canada; 13 Department of Biology, University of Florida, Gainesville, FL, 32607, USA; 14 Department of Geography, University of Wisconsin, Madison, WI, 53706, USA; 15 Department of Forest Ecology & Management, Swedish University of Agricultural Sciences, Umeå 901 83, Sweden; 16 Department of Geography, Kansas State University, Manhattan, KS, 66506, USA; 17 Department of Biology, University of Copenhagen, Copenhagen Ø, 2100, Denmark; 18 Faculdade UnB Planaltina, Universidade de Brasília, Brasília, 73345-010, Brazil; 19 Departamento de Biologia Vegetal, Instituto de Biologia, Universidade Estadual de Campinas, Campinas, 13083-862, Brazil; 20 Forest and Rangeland Ecosystem Science Center, US Geological Survey, Corvallis, OR, 97331, USA; 21 Universidad Nacional de la Patagonia Austral-INTA-CONICET, Río Gallegos, Santa Cruz, 9400, Argentina; 22 Coordenação de Dinâmica Ambiental, Instituto Nacional de Pesquisas da Amazonia, Manaus, 69011, Brazil; 23 Department of Terrestrial Ecosystem Research, University of Vienna, Vienna, 1090, Austria; 24 Environmental Research Institute, University of Waikato, Hamilton, 3240, New Zealand; 25 Landcare Research, Hamilton, 3240, New Zealand; 26 Smithsonian Tropical Research Institute, Balboa, Ancón, Republic of Panama; 27 DBPVA, Centro de Ciências Agrárias, Universidade Federal de São Carlos, Araras, SP, 13600-970, Brazil; 28 Biogéochimie des Ecosystèmes Forestiers, INRA Nancy, Champenoux, 54280, France

## Abstract

Quantifying global patterns of terrestrial nitrogen (N) cycling is central to predicting future patterns of primary productivity, carbon sequestration, nutrient fluxes to aquatic systems, and climate forcing. With limited direct measures of soil N cycling at the global scale, syntheses of the ^15^N:^14^N ratio of soil organic matter across climate gradients provide key insights into understanding global patterns of N cycling. In synthesizing data from over 6000 soil samples, we show strong global relationships among soil N isotopes, mean annual temperature (MAT), mean annual precipitation (MAP), and the concentrations of organic carbon and clay in soil. In both hot ecosystems and dry ecosystems, soil organic matter was more enriched in ^15^N than in corresponding cold ecosystems or wet ecosystems. Below a MAT of 9.8°C, soil δ^15^N was invariant with MAT. At the global scale, soil organic C concentrations also declined with increasing MAT and decreasing MAP. After standardizing for variation among mineral soils in soil C and clay concentrations, soil δ^15^N showed no consistent trends across global climate and latitudinal gradients. Our analyses could place new constraints on interpretations of patterns of ecosystem N cycling and global budgets of gaseous N loss.

Quantifying global patterns of terrestrial nitrogen (N) cycling is central to predicting future patterns of primary productivity, carbon sequestration, nutrient fluxes to aquatic systems, and climate forcing^1^,^2^,^3^,^4^. With limited direct measurements of soil N cycling at the global scale, past syntheses of the ^15^N:^14^N ratio of soil organic matter (represented as δ^15^N relative to a standard) have inferred that hotter and drier ecosystems tend to lose a greater proportion of their N through gaseous pathways^5^,^6^. Global soil δ^15^N patterns are also among the main evidence used to support the idea that plant productivity in tropical ecosystems is less N limited than in temperate ecosystems^6^,^7^. These conclusions have assumed that soils with high δ^15^N lose a greater proportion of N through strongly ^15^N-discriminating loss processes such as NH_3_ volatilization and denitrification rather than through less discriminating loss pathways such as dissolved organic N and NO_3_^−^ leaching^5^,^8^,^9^,^10^.

Past analyses of global soil δ^15^N patterns have examined relatively simple direct relationships between soil δ^15^N and climate or latitude^5^,^6^,^11^. Yet, other factors that might affect soil δ^15^N and co-vary with climate could influence global relationships between soil δ^15^N and climate. For example, a large proportion of the global variation in foliar δ^15^N is explained by the N concentrations of leaves^12^, most likely because plants that have high foliar N concentrations are more likely to be growing in soils where fractionating loss pathways dominate N losses. Like leaves, soil C or N concentrations might be important covariates for soil δ^15^N. Although rates of microbial processing of plant litter are known to vary across climate gradients^13^,^14^,^15^, the extent to which soil C or N concentrations might shape geographic patterns of soil δ^15^N remains unexplored. Soils with different C or N concentrations might consistently vary in their δ^15^N due to differences in the composition of organic matter inputs or rates of microbial processing of organic matter, both of which could affect the N isotopic composition of soil organic matter. In addition, soil texture has the potential to affect the relative importance of different loss pathways^16^ and/or the differential retention of ^15^N-enriched organic matter^17^,^18^,^19^,^20^,^21^. With highly weathered tropical sites more likely to have greater clay concentrations than many high-latitude ecosystems^22^, this may be an additional confounding influence on global patterns of soil δ^15^N.

Beyond improving models of the controls on soil δ^15^N, it is imperative to continue to expand databases of soil δ^15^N because non-linear relationships may become apparent as data on soil ^15^N accumulate, which may affect interpolation of N cycling rates in poorly characterized ecosystems. For example, at the global scale, foliar δ^15^N increases with increasing foliar N concentrations, but only above a mean annual temperature (MAT) of −0.5°C^12^. Such observations are important, because they prompt mechanistic hypotheses that test critical understanding how climate influences ecosystem N cycling. For soil δ^15^N, past syntheses of soil δ^15^N included too few samples, especially for cold ecosystems, to adequately determine whether non-linear relationships exist between soil δ^15^N and climate.

To better understand global patterns of soil δ^15^N, we assembled a global dataset of published and original surface soil δ^15^N values and examined relationships of soil δ^15^N with climate, soil organic C and N concentrations ([C], [N]), and soil clay concentrations. The dataset comprised 5824 measurements of δ^15^N of soil organic matter from surface (<30 cm) mineral soils. Data for an additional 973 organic soils were compiled, but are only analyzed secondarily here as δ^15^N signatures of organic soils are more likely to represent the signatures of plants than the ultimate decomposition products of plant biomass. When averaged at 0.1° latitude and longitude, soils represented 910 locations (Fig. 1) that spanned 44°C (MAT) (−14°C to 30°C) and over 9000 mm mean annual precipitation (MAP) (84–9510 mm) (Fig. 2).

## Results

**Figure 3:**
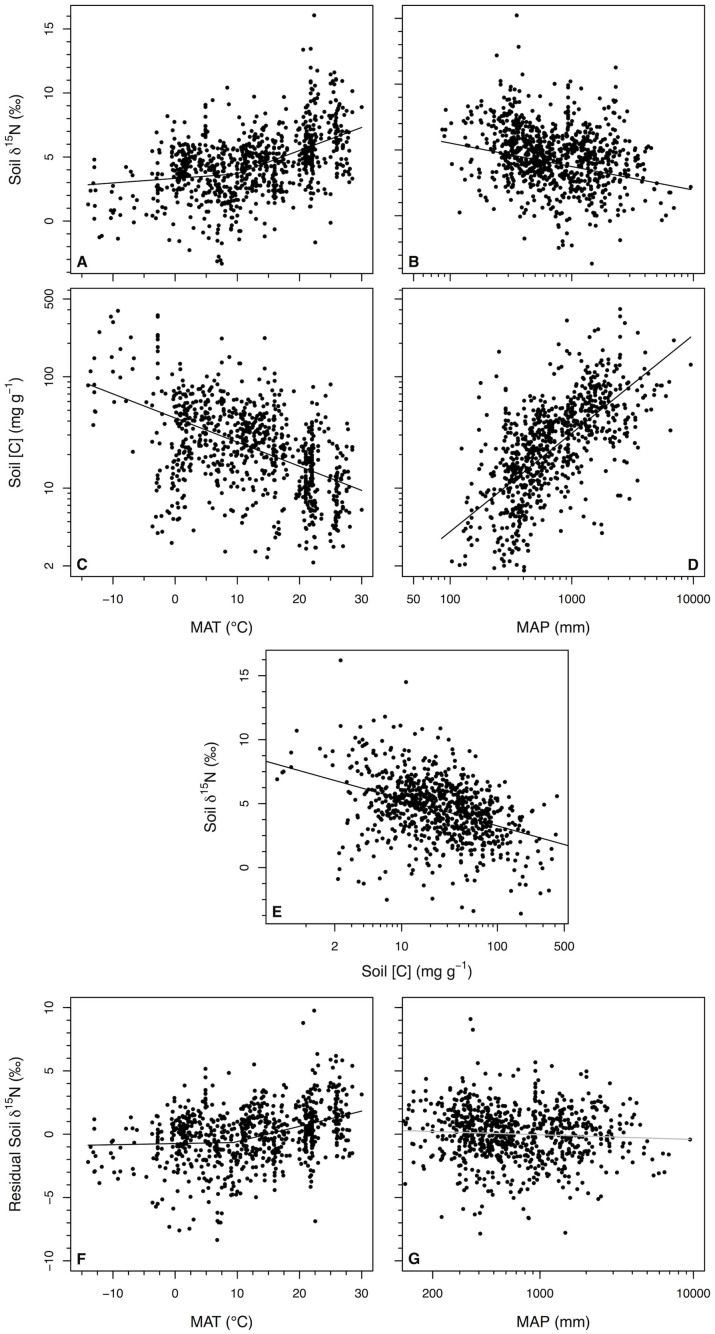
Relationships among climate and soil parameters. Relationships between mean annual temperature (MAT) and mean annual precipitation (MAP) and (A,B) soil δ^15^N (n = 910) and (C,D) soil [C] of surface soils (n = 828). Each point represents values for all samples averaged per 0.1° latitude and longitude. The relationship between soil [C] and soil δ^15^N is shown in panel (E) (n = 828). After accounting for the variation in soil δ^15^N explained by soil [C] (n = 828), the residual variation in soil δ^15^N is shown vs. (F) MAT and (G) MAP (n = 828). Gray regression line is not significant at *P* *>* 0.05. Values displayed in relationships with MAT and MAP were corrected for variation that could be explained by the other climate variable and soil depth.

Congruent with previous research, soil organic matter was more enriched in ^15^N in both hot ecosystems and dry ecosystems than corresponding cold ecosystems or wet ecosystems (Fig. 3; Table 1). Soil δ^15^N increased with increasing MAT at a rate of 0.18 ± 0.02‰ °C^−1^ for soils from ecosystems with MAT > 9.8°C. Yet, below 9.8°C, soil δ^15^N did not change with increasing MAT (0.035 ± 0.024‰ °C^−1^; *P* *>* 0.1). High-precipitation ecosystems had lower soil δ^15^N with soil δ^15^N decreasing at a rate of 1.78 ± 0.24‰ per order of magnitude increase in MAP, after accounting for any co-variation in MAT.

**Figure 4:**
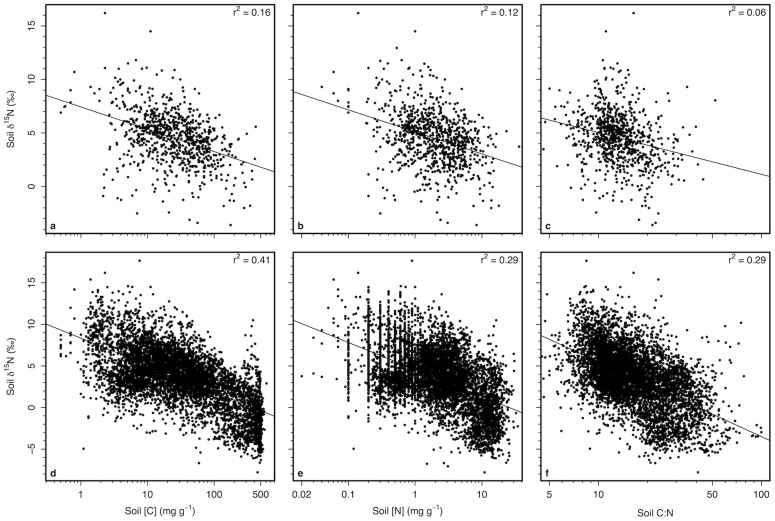
Patterns of soil δ^15^N with soil C and N concentrations. Relationships between (A,D) soil carbon concentrations, (B,E) soil nitrogen concentrations, and (C,F) soil C:N with soil δ^15^N for (A–C) mineral soils and (D–F) mineral as well as organic soil horizons. For (A–C), each point represents soils averaged for 0.1° latitude and longitude. All relationships significant at *P* < 0.001.

At the global scale, soil [C] also declined with increasing MAT and decreasing MAP (*P* < 0.001; r^2^ = 0.42; Fig. 3). Soil δ^15^N was highest for low-C soils and decreased with increasing soil [C] (r^2^ = 0.16, *P* < 0.001, n = 828; Fig. 3, Fig. 4). After accounting for variation among mineral soils in soil [C], the global range in MAT observed here was associated with just 2.7‰ variation in soil δ^15^N (Fig. 3), with MAT explaining much less variation in residual soil δ^15^N (*P* < 0.001, r^2^ = 0.14, n = 828). Likewise, after calculating the residuals between soil δ^15^N and [C], MAP no longer predicted variation in soil δ^15^N (*P* = 0.1). Soil [N] and C:N were weaker predictors of soil δ^15^N than soil [C], but generate similar patterns as soil [C] (Fig. 4).

**Figure 5:**
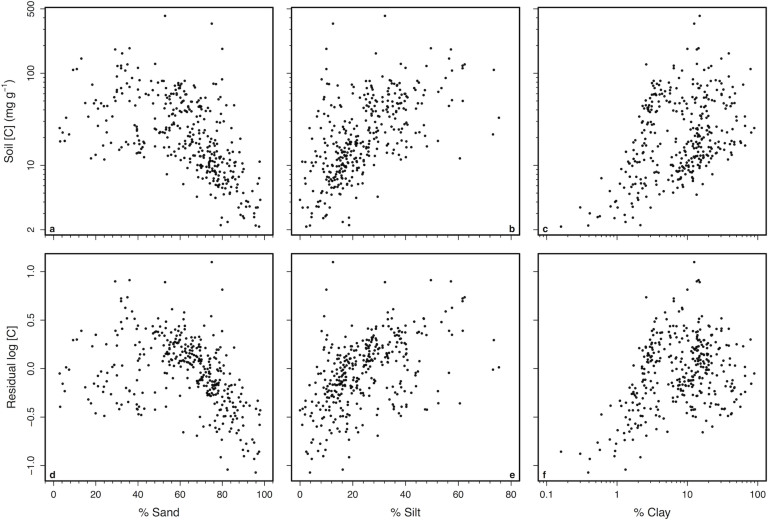
Relationships between soil carbon concentrations and texture. Shown are the percentages of (A,D) sand, (B,E) silt, and (C,F) clay vs. (A–C) soil carbon (mg g^−1^) as well as (D–F) residual log_10_-transformed soil carbon (mg g^−1^) after accounting for MAT, log_10_MAP, and average depth. All points represent soil values averaged to 0.1° latitude and longitude.

**Figure 6:**
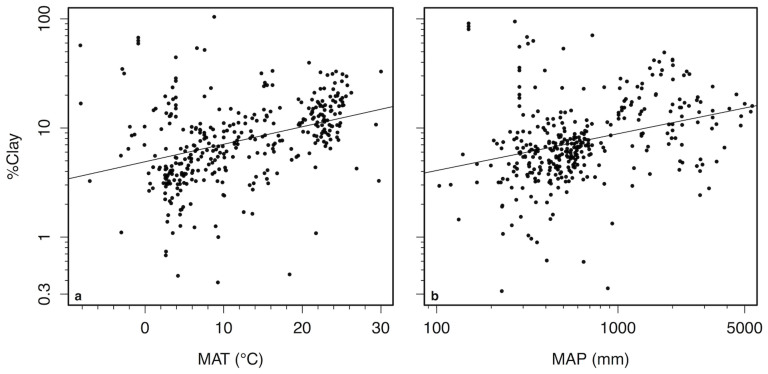
Patterns of soil clay concentrations with climate. Relationships between (A) mean annual temperature (MAT) and (B) mean annual precipitation (MAP) vs. clay concentrations of surface mineral soils. log (%Clay) = −0.84 + 0.016 × MAT + 0.55 × log(MAP); r^2^ = 0.47, *P* < 0.001, n = 359. All points represent soil values averaged to 0.1° latitude and longitude.

**Figure 7:**
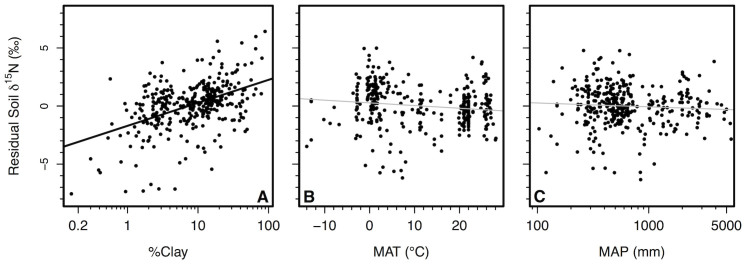
Lack of relationship between climate and soil δ^15^N after accounting for variation in clay concentrations. Relationship between (A) clay concentrations in soil and residual soil δ^15^N after accounting for the relationship between soil δ^15^N and soil [C] (y = 1.71 + 2.01 × log(x), r^2^ = 0.22, *P* < 0.001; n = 355). Also shown are relationships between (B) mean annual temperature (MAT), (C) mean annual precipitation (MAP) and residual soil δ^15^N after accounting for variation in soil [C], clay concentrations, and soil depth. Non-significant relationships are shown in gray. All points represent soil values averaged to 0.1° latitude and longitude.

**Figure 8:**
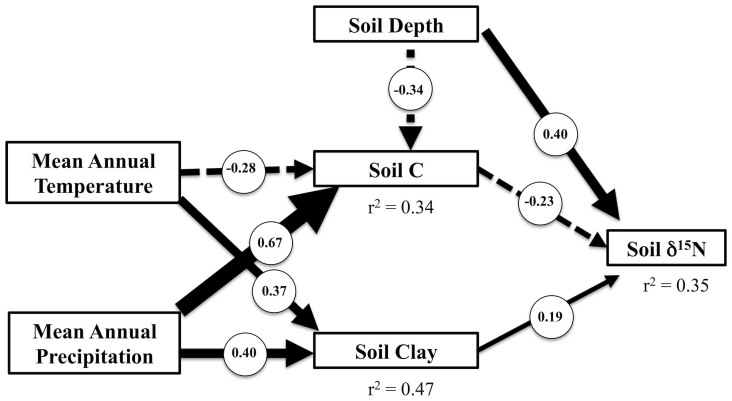
Structural equation model of direct and indirect effects of climate on soil δ^15^N. Path-diagram of final structural equation model containing only significant relationships. Positive coefficients overlaid on solid paths and negative coefficients on dashed paths. Arrow thickness proportional to path coefficient. Direct effects of climate on soil δ^15^N were not significant.

The variation in soil δ^15^N explained by MAT after taking into account relationships with soil [C] could be caused by the high concentrations of clay found in many tropical soils. 30% of the residual variation in soil [C] after accounting for variation in climate could be explained by soil texture (Fig. 5). Soils with greater silt and clay concentrations had greater [C] than sandy soils (Fig. 5). Within our data, hot sites also had greater clay concentrations than cold sites (Fig. 6). After taking into account the positive relationship between clay concentrations and soil δ^15^N (Fig. 7), MAT had no remaining influence on soil δ^15^N (Fig. 7). Interpretations of these sequential regression results were further supported by a structural equation model that simultaneously evaluated both direct effects of climate on soil δ^15^N as well as indirect effects via effects on soil [C] and clay concentrations (Fig. 8). Mean annual temperature and precipitation only influenced soil δ^15^N indirectly through their effects on soil [C] and clay. Direct relationships between climate and soil δ^15^N were not significant (Fig. 8).

## Discussion

The global-scale relationships between soil δ^15^N and both soil [C] and clay suggest that the relationship between soil δ^15^N and climate is indirect, and mediated through climatic effects on soil [C] and clay. Our analyses indicate that known dependencies of microbial processing of soil C and N on temperature and moisture that have been observed experimentally, within soil profiles, and at local to regional scales^23^,^24^,^25^,^26^ largely converge in their effects on soil δ^15^N at the global scale. Further understanding of global soil δ^15^N patterns will require direct quantification of the relative importance of different loss pathways as well as the δ^15^N values and relative quantities of different soil fractions across broad gradients. Among ecosystems, SOM δ^15^N has the potential to be influenced by variation in the ^15^N signature of atmospheric N deposition. Yet, with the signatures of deposited N often near 0‰^9^,^27^, the lack of pattern in SOM δ^15^N along climate gradients once the degree of decomposition and clay are taken into account is unlikely to be driven by specific regions of the world receiving greater amounts of deposited N.

**Figure 9:**
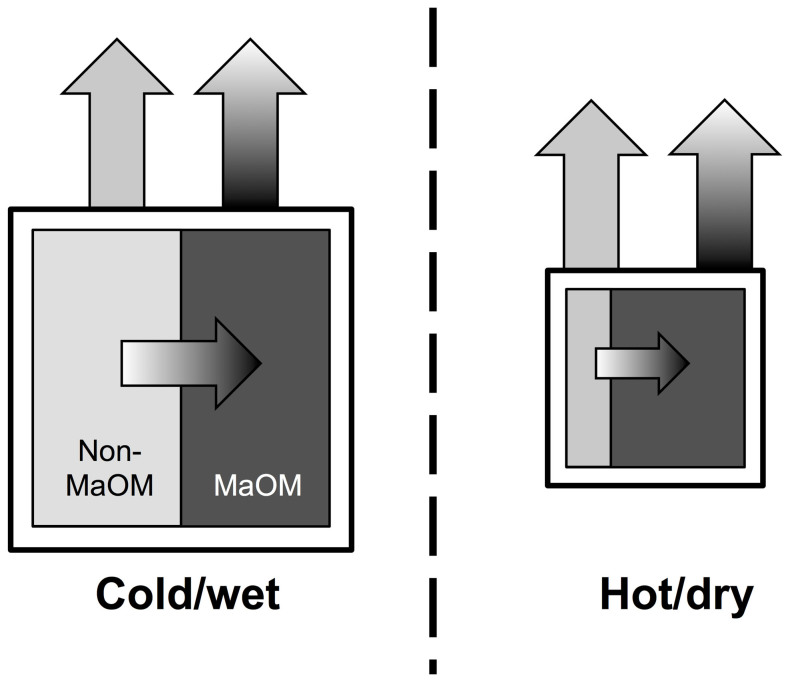
Conceptual model of a hypothesis to explain the higher δ^15^N of soils from hot and/or dry ecosystems. Hot and/or dry ecosystems might not have a greater proportion N lost to fractionating (gradient arrow) vs. non-fractionating (solid arrow) losses. Note, arrows not to scale. Instead, as a result of having organic matter that has been decomposed to a greater degree and/or greater clay concentrations, soils from hot and/or dry ecosystems might simply have a greater proportion of their N in mineral-associated organic matter, which is enriched in ^15^N relative to non-mineral-associated organic matter.

In all, the global patterns of soil δ^15^N revealed here support the hypothesis that hot and/or dry ecosystems might not lose a greater proportion of N to the atmosphere relative to other ecosystems, as has been previously concluded from soil N isotope data^5^,^6^. Although the relationships between clay and soil δ^15^N could be driven by greater proportions of fractionating gaseous N loss in soils with high clay concentrations, an alternative explanation is the relative proportion of fractionating N loss is not directly influenced by clays, but instead is due to clays stabilizing more ^15^N-enriched soil organic matter^17^,^18^,^19^,^20^,^21^. As a result of having greater decomposition of organic matter and/or greater concentrations of clay, it is possible that soils in hotter and/or drier ecosystems have soil organic matter with high δ^15^N as a result of having a greater proportion of their N contained in mineral-associated organic matter as opposed to having a greater proportion of N being lost to fractionating pathways (Fig. 9).

In support of the idea that the proportion of N lost via fractionating pathways might not vary predictably across global climate gradients, recent research has revealed underappreciated amounts of N loss to the atmosphere and aquatic ecosystems suggestive of similar proportions of fractionating N losses across climatic gradients. For example, although tundra ecosystems are typically considered dominated by organic N cycling with little net N mineralization^28^, gross N mineralization rates and N_2_O fluxes can contribute to a high proportion of losses, especially given seasonal asynchronies between mineralization and uptake^29^,^30^,^31^. Tropical ecosystems may lose a larger quantity of N to the atmosphere than many temperate ecosystems. Yet, NO_3_^−^ fluxes in tropical streams can also be more than an order of magnitude greater than temperate streams^32^,^33^. These examples from geographically disparate ecosystems may reflect similar, proportional fractionating losses of N to the atmosphere across latitudinal gradients. Higher N availability that is characteristic of many low-latitude ecosystems may in fact reduce the proportion of N lost in gaseous forms relative to leaching^34^.

Ultimately, future interpretations of global patterns of N cycling will depend strongly on understanding the mechanisms that drive global relationships between clay content, soil C concentrations and soil organic matter δ^15^N. From these relationships, we hypothesized that global patterns of soil δ^15^N may reflect the degree to which soil organic matter has been enriched in ^15^N by microbial processing and protected by mineral association. This would parallel the δ^15^N of soil organic matter consistently increasing with depth across soils globally^25^, which can be attributed to greater degrees of processing of soil organic matter at depth^35^,^36^,^37^. Recognizing the influence of the degree of microbial processing and protection on soil δ^15^N may potentially alter assumptions that soil δ^15^N scales positively with the proportion of N lost via gaseous processes, which has been central to estimates of global denitrification and N_2_-fixation^9^,^38^. Clarifying whether such assumptions are valid is critical. In addition, global patterns of soil δ^15^N need to be rectified with global patterns of plant δ^15^N. For example, it is poorly understood how the relatively high tissue δ^15^N of plants in hot, dry places^12^ relates to our finding that high soil δ^15^N in these locations is best explained by a high degree of decomposition and protection of organic matter. More research is also needed to understand the mechanisms that explain the different inflection points in plant and soil δ^15^N relationships with mean annual temperature, as well as why plants are generally depleted in ^15^N relative to soils^12^. With geographic gradients able to provide a surrogate for future climates^39^,^40^,^41^, interpretations of global patterns of soil δ^15^N will factor prominently in predicting changes in patterns of gaseous N flux to the atmosphere in response to changes in the Earth-climate system.

## Methods

Data on soil δ^15^N were acquired from the literature and by contacting individual researchers known to have collected soil nitrogen isotope data in the past. For each soil, we collected data on geographic coordinates with climate data taken from the original source or 50-year climatic means (1950–2000) were acquired from WorldClim^42^. For each soil sample, we recorded soil depth, soil δ^15^N, and where possible [C], [N], ratio of C to N on a mass basis, and soil texture (percentages of sand, silt, and clay). Due to correlations among soil texture categories, only clay concentrations are included in models here.

Only soils considered by the authors as mineral soils (no litter or O horizons) with depths that averaged <30 cm were included in the main synthesis. Select permafrost samples from below this zone were also included, in order to improve the representation of cold sites as there should be little further processing of soil organic matter once frozen. Subsequent analyses also examined patterns across mineral and organic soil horizons. Soils that were under crops were not included in the synthesis.

To reduce over-representation of individual sites, average soil δ^15^N were derived for each 0.1° latitude and longitude. Average soil δ^15^N was derived by calculating the average δ^15^N for each soil depth and then weighting the average soil δ^15^N by N content if multiple depths were measured. To examine relationships between soil δ^15^N and climate, a non-linear model was used to predict soil δ^15^N with MAT, log-transformed MAP, and average soil depth, which included an independently-fit breakpoint for the relationship between MAT and soil δ^15^N^12^. Subsequent models were used to predict the residuals of the relationship between soil δ^15^N and [C]. A structural equation model (SEM)^43^ was used to test the relative importance of direct vs. indirect linkages between climate and soil δ^15^N. A non-hierarchical model was first developed and then downward stepwise selection was used to generate a hierarchical model that included only significant paths. SEM statistics were calculated in IBM SPSS AMOS version 20.0.0.1 (AMOS Development Corp. Meadville, Pennsylvania, USA). All other statistics were computed in JMP 10.0.2 (SAS Institute, Cary, North Carolina, USA).

## Author Contributions

J.M.C. designed research and created all figures. J.M.C. and L.W. analysed the data, J.M.C. led the writing of the paper with substantial input from A.J.E., L.A., W.T.B., E.N.J.B., M.D.C., N.J.H., E.A.H., A.K., K.K., J.M.K., M.C.M., E.M.S., J.R.M., K.K.M., A.M., G.B.N., R.S.O., S.S.P., P.L.P., C.A.Q., A.R., L.A.S., B.A.S., B.L.T., R.A.G.V., W.W., L.W. and B.Z.

## Figures and Tables

**Figure 1:**
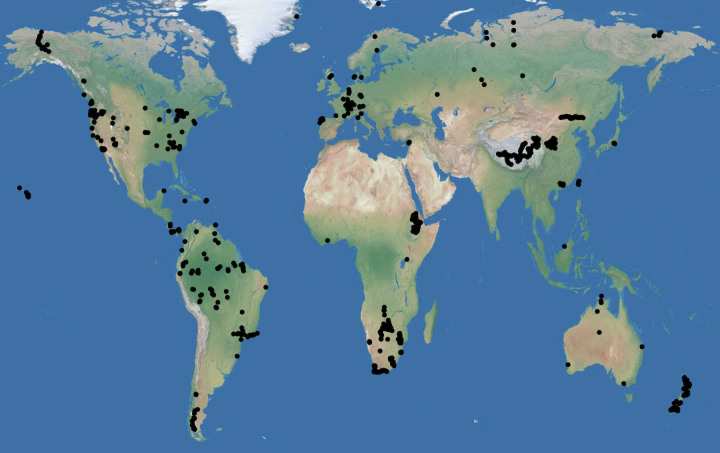
Map of sites used in this study. Map created in JMP 10.0.2.

**Figure 2:**
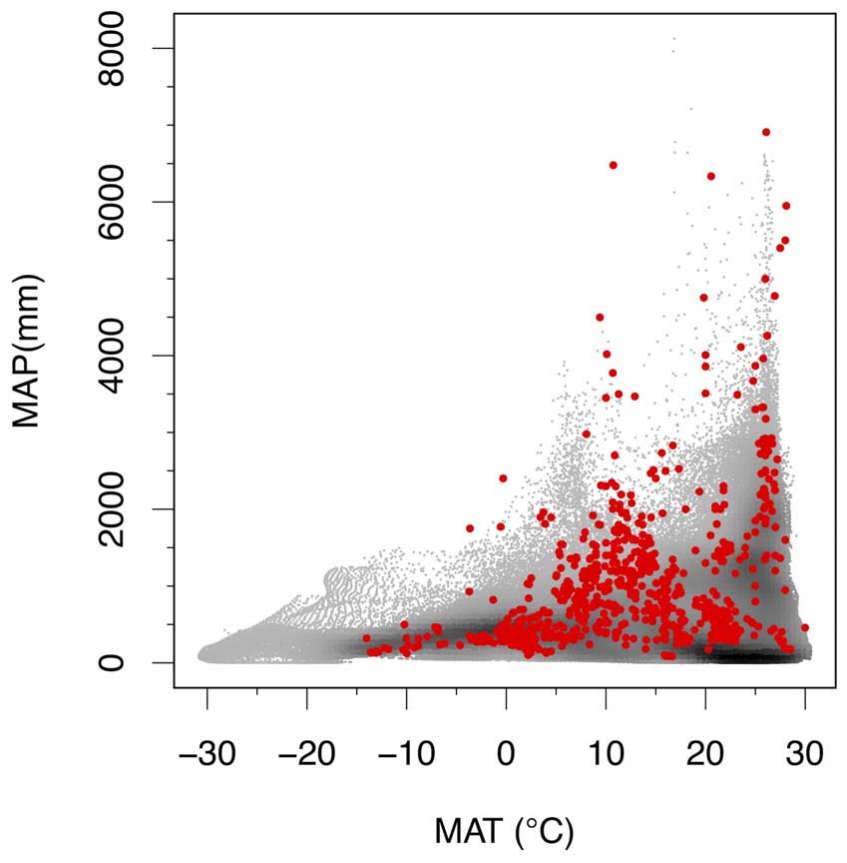
Map of climate space of sites in this study to global terrestrial climate density. Sites used in this study are red. Background points represent the density of ice-free land surface area at particular combinations of mean annual temperature (MAT) and mean annual precipitation (MAP).

**Table 1:** Regression results for mineral soil δ^15^N vs. climate. Inflection point for MAT in the breakpoint model was 9.83 ± 1.83 °C. r^2^ = 0.26 for the breakpoint model and 0.24 for the linear model. Units for MAP were mm before log-transformation

	Estimate	*P*
Breakpoint		
Intercept (‰)	7.71 ± 0.65	<0.001
MAT_Cold_ (‰ °C^−1^)	0.03 ± 0.02	>0.1
MAT_Hot_ (‰ °C^−1^)	0.18 ± 0.02	<0.001
log_10_ MAP (‰)	−1.78 ± 0.24	<0.001
Average Depth (‰ cm^−1^)	0.08 ± 0.02	<0.001
Linear		
Intercept (‰)	8.31 ± 0.64	<0.001
MAT (‰ °C^−1^)	0.12 ± 0.01	<0.001
log_10_ MAP (‰)	−2.10 ± 0.23	<0.001
Average Depth (‰ cm^−1^)	0.09 ± 0.02	<0.001
